# A population-specific reference panel for improved genotype imputation in African Americans

**DOI:** 10.1038/s42003-021-02777-9

**Published:** 2021-11-05

**Authors:** Jared O’Connell, Taedong Yun, Meghan Moreno, Helen Li, Nadia Litterman, Alexey Kolesnikov, Elizabeth Noblin, Pi-Chuan Chang, Anjali Shastri, Elizabeth H. Dorfman, Suyash Shringarpure, 23andMe Research Team, Stella Aslibekyan, Elizabeth Babalola, Robert K. Bell, Jessica Bielenberg, Katarzyna Bryc, Emily Bullis, Daniella Coker, Gabriel Cuellar Partida, Devika Dhamija, Sayantan Das, Sarah L. Elson, Teresa Filshtein, Kipper Fletez-Brant, Pierre Fontanillas, Will Freyman, Pooja M. Gandhi, Karl Heilbron, Alejandro Hernandez, Barry Hicks, David A. Hinds, Ethan M. Jewett, Yunxuan Jiang, Katelyn Kukar, Keng-Han Lin, Maya Lowe, Jey McCreight, Matthew H. McIntyre, Steven J. Micheletti, Joanna L. Mountain, Priyanka Nandakumar, Aaron A. Petrakovitz, G. David Poznik, Morgan Schumacher, Janie F. Shelton, Jingchunzi Shi, Christophe Toukam Tchakouté, Vinh Tran, Joyce Y. Tung, Xin Wang, Wei Wang, Catherine H. Weldon, Peter Wilton, Corinna Wong, Adam Auton, Andrew Carroll, Cory Y. McLean

**Affiliations:** 1 23andMe, Inc., Sunnyvale, CA USA; 2 Google Health, Cambridge, MA USA; 3 Google Health, Palo Alto, CA USA

**Keywords:** Genome-wide association studies, Haplotypes

## Abstract

There is currently a dearth of accessible whole genome sequencing (WGS) data for individuals residing in the Americas with Sub-Saharan African ancestry. We generated whole genome sequencing data at intermediate (15×) coverage for 2,294 individuals with large amounts of Sub-Saharan African ancestry, predominantly Atlantic African admixed with varying amounts of European and American ancestry. We performed extensive comparisons of variant callers, phasing algorithms, and variant filtration on these data to construct a high quality imputation panel containing data from 2,269 unrelated individuals. With the exception of the TOPMed imputation server (which notably cannot be downloaded), our panel substantially outperformed other available panels when imputing African American individuals. The raw sequencing data, variant calls and imputation panel for this cohort are all freely available via dbGaP and should prove an invaluable resource for further study of admixed African genetics.

## Abstract (web summary)

O’Connell et al. construct a new genome-wide imputation reference panel comprising 2,269 individuals of Sub-Saharan African ancestries. They adapt DeepVariant to create best practices for reference panel development and generate a high quality, publicly available resource that will further empower high resolution genome-wide imputation efforts in individuals of African ancestries.

## Introduction

Genome-wide association studies (GWAS) have greatly improved our understanding of human genetics over the past decade. To date, GWAS have largely been conducted in cohorts of European descent, leaving the genetic architecture of complex traits in non-Europeans underexplored^1,2^. A crucial component of GWAS is genotype imputation^3^, which requires a large reference panel of sequenced individuals with similar ancestry to the cohort being studied. Existing publicly available panels are predominantly composed of individuals of European descent. For example, the public release of the Haplotype Reference Consortium (HRC)^4^ panel consists of 27,166 individuals who are largely of European descent, except for 2001 individuals included from the 1000 Genomes Project (1KGP)^5^, only 661 of whom have substantial African ancestry. There are two imputation panels that focus on African genomic content: the Consortium on Asthma among African-ancestry Populations in the Americas (CAAPA)^6^ and the African Genome Resources (AGR) panel^7^. CAAPA contains individuals with African ancestry residing in the Americas and some Atlantic African individuals, making it very relevant for imputing African Americans (AFAMs) but it is a relatively small panel (*N* = 883). AGR has limited data from Atlantic African individuals, making it less appropriate for imputation of AFAM individuals. The recently available TOPMed^8^ imputation server provides imputation with substantially more individuals with African ancestry (over 20,000 individuals^9^) but the TOPMed panel is not downloadable due to consent restrictions, limiting its utility to data that can be uploaded for imputation. A full (to our knowledge) list of imputation panels with African content is available in Supplementary Table 1.

This biased reference panel composition generally leads to substantially poorer imputation quality for non-Europeans relative to Europeans. To help remedy this situation, we introduce an AFAM reference panel, composed of 2,269 American individuals with high amounts of (mainly Atlantic) African ancestry sequenced at ~15× coverage. We evaluated multiple single-sample variant callers, joint genotyping methods, and imputation panel creation methods, and ultimately generated an optimized reference panel using DeepVariant for single-sample calling, GLnexus for joint calling, and SHAPEIT-4 for genotype phasing. The optimized reference panel contains 45,802,366 single-nucleotide polymorphisms (SNPs) and 9,160,064 indels, after excluding all singleton variants. Many of the remaining SNP and indel calls are not present in publicly available panels such as 1KGP/HRC and impute well. This reference panel substantially improves imputation accuracy for individuals with Atlantic African ancestry compared to other publicly available panels, in particular for lower frequency variants. The panel and its associated sequencing data are publicly available on the database of Genotypes and Phenotypes (dbGaP) (study accession: phs001798.v2.p2).

## Results

### A reference panel enriched for haplotypes derived from Atlantic Africa

We re-contacted 71,455 customers who met the following criteria: had consented to participate in 23andMe research, identified as having African ancestry, were over 18 years of age, joined 23andMe after 2010, had answered >100 survey questions, and who were residing in the United States. From this pool of re-contacted candidates, 5,404 individuals further consented to have their individual-level sequencing data made available via dbGaP. We then sequenced the 2,294 individuals with the highest amount of estimated Sub-Saharan African ancestry to produce the final cohort. Finally, we uploaded their sequence data to dbGaP after removing quality control (QC) failures. Sequencing was performed to an average aligned coverage of 14.8× (Supplementary Fig. 1). After pruning close relatives (see “Methods”), there were 2,269 unrelated samples in the final imputation panel, hereafter denoted as the “AFAM panel”.

Country of birth was reported for 1,853 members of the AFAM panel. Of these, the majority were born in the United States (91.7%), with a small number of individuals from Caribbean countries (4%), Africa (2%), and Europe (1.7%), and fewer than five individuals from each of Canada, Asia, South America, and Oceania (Supplementary Table 2). Hence, although the vast majority of this cohort were born in the Americas, small numbers of individuals were not; this is corroborated by ancestry analysis in the next section, which highlights some small clusters of non-admixed individuals.

**Fig. 1:**
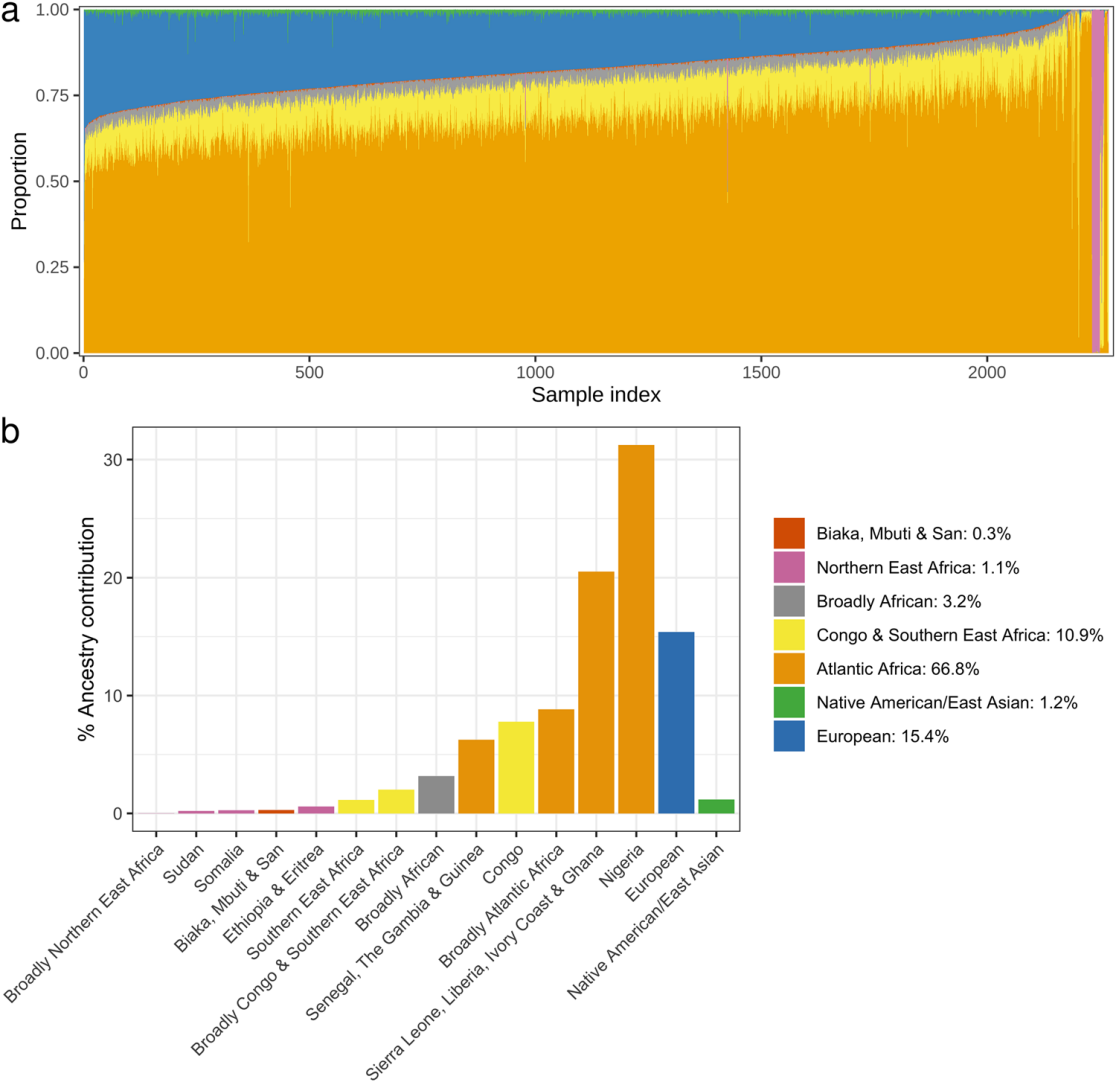
The ancestry composition of the AFAM panel. **a** Estimated ancestry proportions for each of the 2,269 sequenced individuals in the AFAM panel. Only African regions and other regions that contributed substantially to admixture were included. Each column represents an individual colored by their respective estimated ancestry. Columns were ordered first by cluster membership identified in Supplementary Fig. 2 and second by proportion of African ancestry. To ensure anonymity, each individual’s predicted ancestry proportions were multiplied by random numbers drawn uniformly from [0.9, 1.1] and the resulting values were normalized to sum to 1. **b** Average percentage of ancestry contribution across the entire AFAM panel for 13 different African regions and the two largest non-African admixed contributions (European and Native American/East Asian). Individual ancestry contributions were estimated by the 23andMe ancestry classifier.

The ancestral composition of individuals in the AFAM panel was estimated by the most recent iteration of 23andMe’s local ancestry inference algorithm, which assigns ancestry to short genomic segments of phased genotype microarray data using a support vector machine, followed by smoothing using a Hidden Markov Model^10^. It uses a reference panel containing over 14,000 unadmixed unrelated individuals (including 1,991 African individuals; see Supplementary Table 3) and has been successfully used in previous studies of AFAM ancestry^11,12^. The distribution of ancestry within individuals (Fig. 1a) and aggregated ancestry proportions across the entire cohort (Fig. 1b) show that the majority of individuals have varying degrees of Sub-Saharan African (average 82.3%), European (average 15.4%), and East Asian & Native American (average 1.2%) ancestry. The Sub-Saharan African ancestry was mainly Atlantic African (66.8%) with a substantial contribution from Congo/South East Africa (10.9%). There were also small numbers of individuals with little or no European admixture and of Northern East African descent. This is broadly comparable to previous studies^12^ with some notable outliers that are also highlighted via the dimension reduction and clustering described next.

We performed UMAP (Uniform Manifold Approximation and Projection) dimensionality reduction on the first 15 principal components of the AFAM samples along with six ethnicities from 1KGP as an unsupervised complement to our ancestry classifier^13,14^ (Supplementary Fig. 2). The AFAM samples predominantly cluster with the admixed African Caribbean in Barbados (ACB) and African Ancestry in Southwest US (ASW) populations from 1KGP, although a small number of individuals (~2%) cluster with Yoruba in Ibadan, Nigeria (YRI)/Esan in Nigeria, Mende in Sierra Leone, or Luhya in Webuye, Kenya. Detailed ancestry proportions for manually curated clusters show that the 23andMe ancestry classifier is concordant with this unsupervised technique (Supplementary Table 4).

### Development of an optimized reference panel

Imputation reference panel quality depends on both the breadth of haplotypes represented within the panel and the accuracy of the variants called in the individuals. With a fixed sequencing budget (as was the case in this project), these are competing requirements. Total sequencing cost is driven largely by total sequencing coverage and higher per-individual sequencing coverage produces more accurate variant calls but reduces the number of individuals able to be sequenced. At the ~15× coverage level chosen for this reference panel, we sought to optimize reference panel quality by performing three independent experiments to identify the best-performing single-sample variant caller and joint genotyping method.

**Fig. 2:**
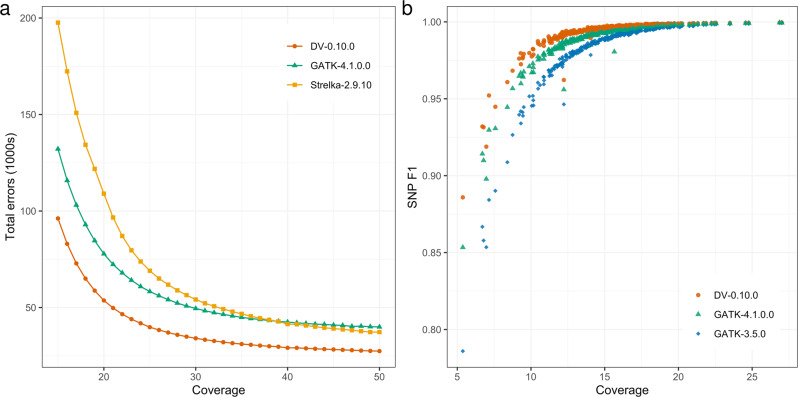
Single-sample variant-calling accuracy as a function of sequence coverage. **a** Total errors (SNP + indel; lower is better) in HG002 in the Genome in a Bottle v4.1 truth regions as a function of sequence coverage for DeepVariant-0.10.0, GATK-4.1.0.0, and Strelka-2.9.10. **b** F1 metric (harmonic mean of precision and recall; higher is better) per sample for SNPs as a function of sequence coverage in a subset of 23andMe AFAM samples (*N* = 292). Each sample produces three points at a single coverage level, indicating the F1 performance of that sample using each of the three variant callers. DeepVariant substantially outperforms both versions of GATK, in particular on lower coverage data.

First, we evaluated single-sample variant-calling accuracy as a function of autosomal sequencing coverage using the well-characterized HG002 sample from NIST Genome in a Bottle^15,16^. Given that GIAB benchmark currently does not include any individual of African ancestry, we used the most extensively characterized and reliable truth set of HG002 for the evaluation of the bioinformatics pipelines. We synthetically downsampled HG002 sequence coverage to all coverages from 15 to 50×, performed variant calling on the downsampled BAM with GATK4^17^, DeepVariant v0.10^18^, and Strelka2^19^, and assessed the resulting variant-calling accuracy in the HG002 v4.1 truth set using hap.py^20^. At all sequence coverages, the total number of errors produced by DeepVariant was lower than either GATK or Strelka2, with a more pronounced impact at lower coverages (Fig. 2a). Notably, DeepVariant at ~21× coverage achieved the same accuracy as 30× samples processed through GATK4, suggesting that DeepVariant can be used to increase accuracy of individual samples in smaller cohorts, or to expand the scale of cohorts while maintaining high accuracy. Further, we found a greater dependency on coverage for Indels vs. SNPs (Supplementary Fig. 3) and observed that lower coverages increase false negative and genotype errors more than false positives (Supplementary Fig. 4).

Second, we evaluated single-sample variant-calling accuracy on a subset of the 23andMe AFAM panel samples (*N* = 292) using truth data carefully curated from a 23andMe microarray containing 387,493 SNPs and 73 indels after stringent quality control (“Methods”). Based on the above downsampling analysis, we evaluated GATK-3.5, GATK-4.1.0.0, and DeepVariant-0.10.0 pipelines for single-sample performance in each of the 292 AFAM samples (Table 1). DeepVariant has substantially higher F1 metrics for both SNPs (Fig. 2b) and indels, with the caveat that there were only a small number of high-quality indels available on our microarray. DeepVariant’s greater F1 score is largely driven by higher sensitivity, with 0.74% and 0.96% higher sensitivity than the next best method (GATK4) for SNPs and indels, respectively (Table 1). Precision was comparable across all three methods. Differences were particularly pronounced at lower coverages; the average SNP F1 metric for samples with coverage between 10× and 15× was 99.0% for DeepVariant vs. 98.1% for GATK4 (Fig. 2b).

**Table 1:** Single-sample variant caller accuracy in 292 23andMe AFAM samples.

Caller	Type	F1	Recall	Precision	TP	FN	FP	FP.gt	FP.al
DV-0.10.0	SNP	**0.993608**	**0.990577**	**0.996658**	60,491,423	575,433	202,836	180,075	5
GATK-3.5.0	SNP	0.983289	0.970674	0.996237	59,275,997	1,790,859	223,902	213,815	5
GATK-4.1.0.0	SNP	0.989730	0.983305	0.996240	60,047,371	1,019,485	226,652	214,521	6
DV-0.10.0	INDEL	**0.988898**	**0.982188**	0.995701	2,316	42	10	5	5
GATK-3.5.0	INDEL	0.975557	0.956319	0.995585	2,255	103	10	10	0
GATK-4.1.0.0	INDEL	0.984338	0.972858	**0.996092**	2,294	64	9	9	0

Truth data were curated from microarray data, which contained predominantly SNPs. Accuracy metrics were computed using hap.py.

Bold cells in the F1, Recall, and Precision columns indicate the best caller performance for that metric in the given variation type.

*DV* DeepVariant, *F1* the harmonic mean of recall and precision, *FN* false negatives, *FP* false positives, *FP.al* allele mismatches, *FP.gt* genotype mismatches, *TP* true positives.

Third, we evaluated the imputation performance of the 23andMe AFAM panels in samples with Atlantic African ancestry. The evaluation samples were all 240 individuals from the populations ASW, ACB, and YRI in 1KGP, who possessed both deep-sequencing data and Illumina Omni2.5 genotype array calls. Imputation was performed with Beagle 5.1^21^ using the publicly available Omni2.5 genotype array calls as input. To fairly evaluate the imputation performance of the candidate panels, we evaluated the imputation results against two publicly available sets of “ground truth” calls, one generated with GATK Best Practices (generated by New York Genome Center, https://www.internationalgenome.org/data-portal/data-collection/30x-grch38) and the other generated with the DeepVariant-GLnexus (DV-GLx) Best Practices optimized pipeline^18,22,23^ (“Methods”) (Supplementary Fig. 5). Imputed genotypes were binned by alternate allele frequency computed in the ground truth 1KGP cohort with all 2,504 samples included and, within each bin, the squared Pearson’s correlation between all imputed genotype dosages and the hard genotypes from the “ground truth” sequencing data was calculated (often referred to as “aggregate *R*^2^”). For these analyses, we treated variants that were present in the truth set but missing from a panel as being imputed to homozygous reference, which penalizes panels with missing variation.

We joint-called the 2,269 unrelated AFAM samples to generate candidate reference panels based on two methods: GATK Best Practices^17^ and DV-GLx^18,22,23^. For quality control, we measured the distributions of read coverage depth, duplication rate, variant call confidence, and transition-transversion ratio, and found that the majority of samples had similar properties (Supplementary Fig. 1). For each of the two joint-called data sets, we first evaluated the impact of genotype refinement and different phasing algorithms on imputation performance, restricted to chromosome 20 only for computational considerations. As the AFAM samples were sequenced at intermediate coverage (~15×), with 5.8% of samples having <10× coverage, we investigated the utility of applying the computationally intensive step of refining genotype likelihoods into discrete genotypes, as was used in low-coverage (~7×) projects such as the 1KGP^5^, UK10K^24^, and HRC^4^ studies. In addition, we compared the relative performance of two state-of-the-art phasing algorithms Eagle-2.4.1^25^ and SHAPEIT-4.1.3^26^. Imputation performance was evaluated for all eight resulting chr20 panels using the 240 1KGP samples described above. For both the GATK and DV-GLx chr20 panels, SHAPEIT-4.1.3 phasing yielded better imputation performance (Supplementary Fig. 6). Notably, the DV-GLx chr20 panels performed better without genotype refinement, whereas the GATK chr20 panels performed better with genotype refinement, although the difference was modest for both callers.

**Fig. 3:**
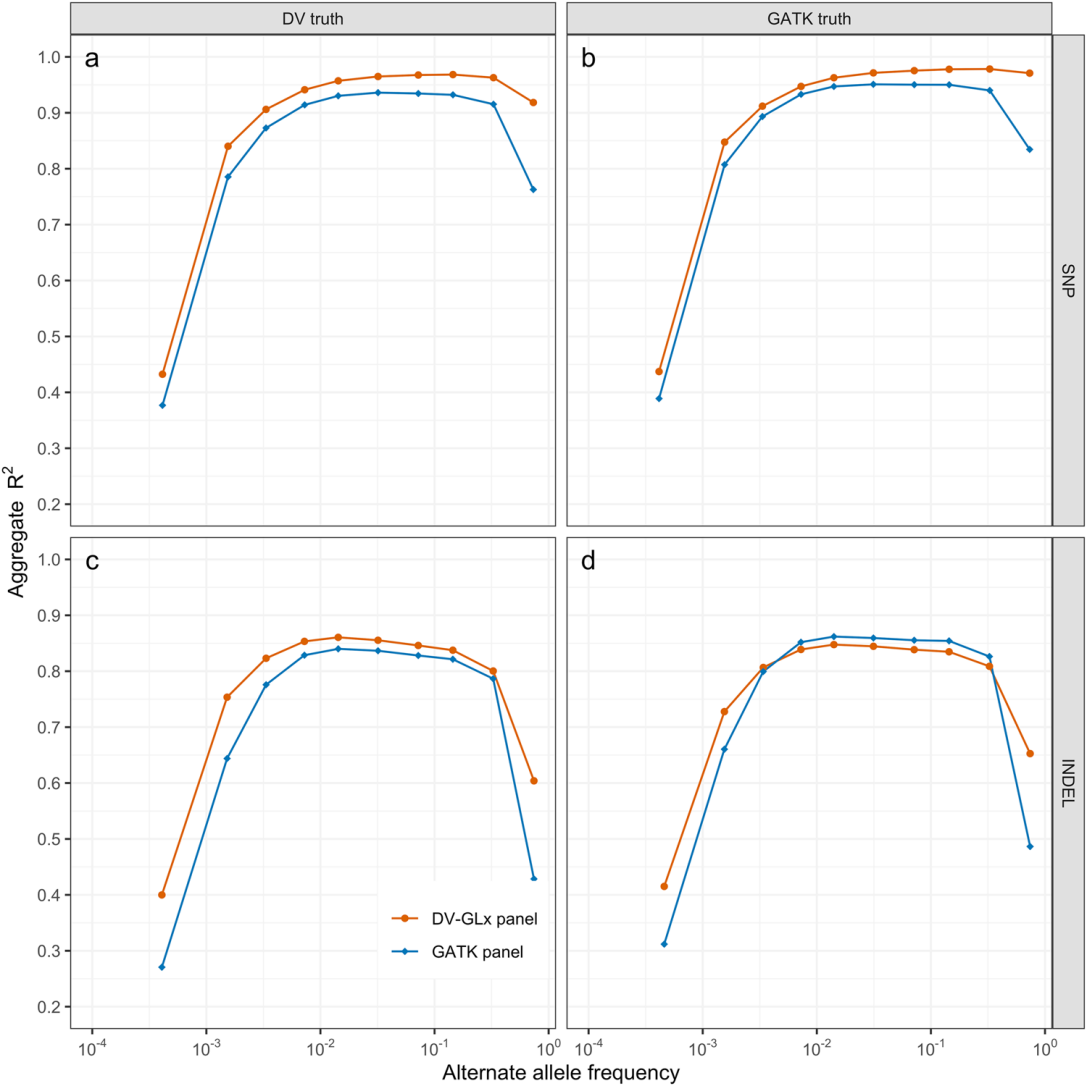
Imputation accuracy of candidate AFAM reference panels with 1KGP individuals of African ancestry. Aggregate *R*^2^ using DeepVariant-GLnexus optimized reference panel (DV panel) and GATK Best Practices (GATK panel) when imputing Illumina HumanOmni 2.5 genotype array calls and evaluating on deeply sequenced (30×) “ground truth.” Variants present in the “ground truth” but missing from the reference panel are imputed as homozygous reference calls, which penalizes panels that have missing variation. **a** SNP *R*^2^ using ground truth generated with DeepVariant + GLnexus (DV truth), **b** SNP *R*^2^ using ground truth generated with GATK Best Practices (GATK truth), **c** Indel *R*^2^ using ground truth generated with DeepVariant + GLnexus, and **d** Indel *R*^2^ using ground truth generated with GATK Best Practices. See also Supplementary Fig. 6.

Based on the chr20 results, we evaluated genome-wide imputation performance for two candidate reference panels: a DV-GLx panel directly phased with SHAPEIT-4.1.3 (hereafter “DV-GLx AFAM panel”) and a GATK panel with genotype likelihoods refined into discrete genotypes using Beagle 4.1^27^ and then phased with SHAPEIT-4.1.3 (hereafter “GATK AFAM panel”). For SNPs, genotypes imputed with the DV-GLx AFAM panel showed higher aggregate *R*^2^ with the ground truth than variants imputed with the GATK AFAM panel, consistently in all allele-frequency bins and regardless of whether the ground truth used was generated with DV-GLx or GATK (Fig. 3a, b). For indels, the results are subtler; the DV-GLx AFAM panel consistently outperformed the GATK AFAM panel when using the DV-GLx ground truth, but when using the GATK ground truth, the GATK AFAM panel achieved better performance in the mid-AF ranges, while the DV-GLx AFAM panel outperformed in the lowest and highest AF bins (Fig. 3c, d).

### Imputation performance relative to existing panels containing African ancestry

**Fig. 4:**
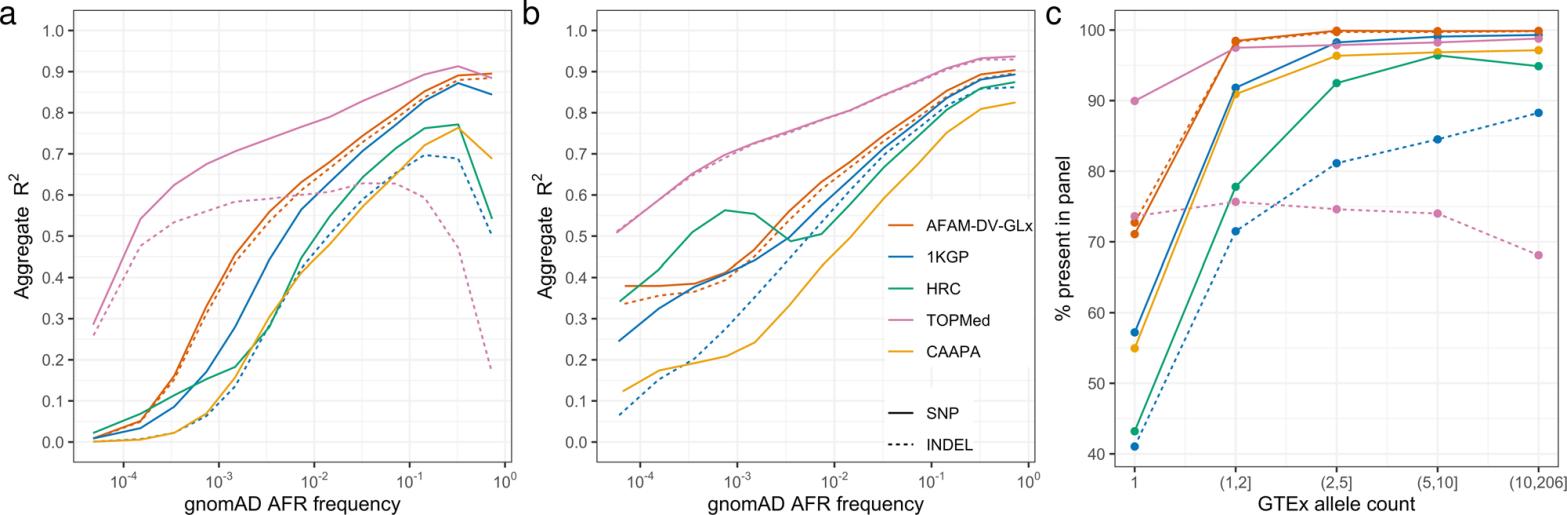
Imputation performance for five different panels using a truth set containing 103 GTEx WGS individuals imputed with an emulated 523 K 23andMe microarray. **a** Aggregate *R*^2^ between the imputed dosages and sequence genotypes as a function of the alternate allele frequency reported for African Americans in the gnomAD r3 data set. We treat variants missing from the panel to be imputed as homozygous reference here, which penalizes panels that have missing variation. **b** Aggregate *R*^2^ for variants only within a given panel (a more lenient measure than in **a**). **c** The proportion of GTEx variants present at different allele counts in each panel. All panels have good sensitivity for SNPs with >2 copies of the allele in GTEx, whereas substantial numbers of indels appear missing from both TOPMed and 1KGP (HRC/CAAPA have no indels). The same legend is shared across all three figure panels.

We further evaluated the DV-GLx AFAM panel imputation performance using a high-coverage whole-genome sequencing (WGS) truth set from 103 individuals in GTEx v8^28^, who identified as AFAM (“Methods”). Microarray genotypes for these individuals were emulated for the current 23andMe microarray from WGS data and were pre-phased with SHAPEIT-4 (510,513 autosomal SNPs). Imputation was then performed with five different reference panels: (1) the DV-GLx AFAM panel (*N* = 2,269), (2) the HRC panel (*N* = 27,165)^4^, (3) the 1KGP phase 3 panel (*N* = 2,504)^5^, (4) the TOPMed panel (*N* = 97,256)^8^, and the CAAPA panel (*N* = 883)^6^. Imputed genotypes were binned by alternate allele frequency taken from the AFAM allele frequency in gnomAD r3^29^. TopMED and CAAPA results used their respective imputation servers, whereas AFAM, HRC, and 1KGP panels were imputed locally using Minimac 4^30^ (the imputation software used by the imputation servers). We calculated aggregate *R*^2^ in two ways: first, treating variants missing from the truth set as being imputed as homozygous reference (Fig. 4a) and, second, only calculating correlation on genotypes from truth variants that intersect each panel (Fig. 4b). The former penalizes panels with putatively missing variation. We also provide overall genotype discordance and non-reference discordance, which produce qualitatively similar results (Supplementary Tables 5 and 6).

As expected, the TOPMed panel produces the strongest results across the allele-frequency spectrum for SNPs, followed by the DV-GLx AFAM panel. For example, consider SNPs at 0.5% allele frequency, TOPMed achieves an aggregate *R*^2^ of 0.75, followed by DV-GLx AFAM (0.59), 1KGP (0.49), HRC (0.35), and CAAPA (0.35), when using the more stringent performance metric (Fig. 4a). Surprisingly, HRC imputation performed worse than 1KGP on these individuals, despite HRC being a superset of 1KGP. This may be due to the more intricate phasing pipeline employed in 1KGP or may be an artifact of the imbalance of ethnicities in HRC. Figure 4b shows the accuracy of each panel when not penalizing missing variation. Accuracies are largely unchanged at higher frequencies (say >1%), suggesting all panels are capturing most common SNPs. Accuracy is substantially higher in Fig. 4b vs. 4a at frequencies lower than ≈0.1% (except for TopMED), highlighting the lack of completeness of the smaller panels at the rarer end of the frequency spectrum.

The performance for indels is more complicated. Due to an apparently large amount of missing indels in the TOPMed panel, it performs worse than DV-GLx AFAM for common indels with frequency approximately >0.5% (Fig. 4a). When evaluating correlation only for variants within a given panel, TOPMed imputation is systematically more accurate across the allele-frequency spectrum (Fig. 4b). Indels are not present in the HRC or CAAPA panels.

We investigated the completeness of variation in each panel by looking at the proportion of alleles in our truth set that were found in each panel, stratified by allele count (Fig. 4c). Singletons were most revealing, with TOPMed containing 90% of singleton SNPs and 74% of singleton indels, followed by AFAM (71% and 73%), 1KGP (57% and 41%), HRC (43% and 0%), and lastly CAAPA (55% and 0%). For SNPs with allele count > 2, both AFAM and TOPMed contained nearly all SNPs (>97%) in the GTEx truth set. Indels in the TOPMed panel appear to have been aggressively filtered, with only 71% the GTEx indels present in the TOPMed panel compared to 91% for AFAM.

## Discussion

Increasing the representation of samples of non-European ancestry in genomic data sets is critical for reducing the potential of polygenic risk scores to exacerbate health disparities^1^ and discovering disease-associated variants specific to non-European samples^31^. The deep human history in Africa results in lower levels of linkage disequilibrium in African populations. Consequently, populations of recent African origin (such as AFAMs) are efficient for identification of causal polymorphisms within a candidate sequence^32^, but further emphasize the need for African haplotypes in imputation reference panels. Here we have introduced an imputation reference panel that is enriched for Atlantic African ancestry as a resource for researchers investigating AFAM genetics. Extensive evaluations of single-sample variant calling showed that DeepVariant consistently outperformed GATK across a spectrum of sequencing coverage on these data. These improvements in single-sample variant calling yielded a modest improvement in downstream imputation performance. In particular, due to greater sensitivity, the DV-GLx reference panel provided a much larger set of variants for association testing than the GATK Best Practices reference panel. When contrasted with the 1KGP, HRC, and CAAPA panels, the DV-GLx panel provided substantially better imputation performance for rarer variants. The TOPMed imputation server yielded far better imputation for SNPs than our panel due to its much larger sample size. However, the TOPMed panel cannot be downloaded due to consent restrictions, so only data consented to be uploaded to a cloud imputation service can take advantage of the large TOPMed panel. The TOPMed indel set also appeared to be very stringently filtered, perhaps at the cost of sensitivity.

Refinement of genotype likelihoods into hard genotypes is a common practice for generating imputation panels from low-coverage sequencing data^4,5,24,33,34^. However, it is a computationally expensive step that introduces substantial complexity into the processing pipeline to parallelize efficiently genomewide (“Methods”). The DV-GLx panels evaluated here showed no performance improvement from genotype refinement, likely owing at least in part to the relatively high accuracy of single-sample DeepVariant calls and well-calibrated genotype likelihood estimates on low-coverage samples, enabling more accurate joint genotyping by GLnexus^22,23^, mitigating the need for further refinement using linkage disequilibrium-based context. This somewhat surprising result further increased the relative computational efficiency to create the DV-GLx AFAM panel compared to the GATK AFAM panel.

Although this study demonstrates the importance of increasing genetic diversity in imputation panels, there are limitations that must be taken into account. Evaluation of imputation panels generated by different variant-calling pipelines is sensitive to selected metrics and the ground truth calls used. Ground truth variants generated using a particular joint-calling method bias result toward imputation panels generated with the same method. Restricting the evaluated sites to those called consistently among all calling pipelines ignores differences in variant detection sensitivity and biases toward easily called variants. To mitigate these issues, we evaluated candidate panel performance on multiple ground truth sets generated using both candidate panel joint-calling methods and binned aggregate *R*^2^ metrics based on allele frequencies computed in an independent data set.

The DV-GLx AFAM imputation panel and related sequencing data are available via NCBI dbGaP. As a standalone imputation panel, it can freely be used to improve imputation in AFAM cohorts. In addition, combining the raw data with other publicly available data such as the recently released high-coverage 1KGP individuals would increase the European and American content, and the resulting multi-ethnic panel would likely lead to even better imputation for underrepresented admixed populations, in particular AFAM and Latino cohorts. We believe that these resources are a valuable contribution to further research of complex trait genetics in non-European populations.

## Methods

### Sample selection and sequencing

The full study was approved by the Ethical & Independent Review Services Institutional Review Board (IRB). Individuals were sequenced to an expected 17 × coverage. Reads were aligned to GRCh38^35^ (including alt contigs) using BWA-MEM^36^ (version 0.7.16a-r1181) and PCR duplicates were marked with Picard (version 2.1.0). As DNA was extracted from saliva, bacterial contamination resulted in an average aligned coverage of 14.8 × with high variation in coverage (Supplementary Fig. 1). We excluded samples with aligned coverage < 3 × or estimated contamination^37^ (from other human DNA) > 5% from downstream analysis. This resulted in 2,294 individuals passing (relatively liberal) single-sample QC.

We estimated robust kinship coefficients and IBD0 proportions using AKT^38,39^. These were used to remove 25 individuals with close relatives (first cousin or nearer) to create a panel of 2,269 unrelated individuals. There were 15 parent–child pairs (including one full trio), three sibling pairs, and seven first cousin (or similar) pairs. Relatedness pruning was simple; children in duos/trios were first excluded (as these can be useful for validation). After this, only familial cliques of size two remained; we chose the higher coverage individual from each clique to maximize data quality. It is noteworthy that although these related individuals are not in the imputation panel, their raw sequencing data are available in dbGaP.

### Evaluation of genotype refinement and phasing of reference panels

Joint-called data sets generated using GATK Best Practices^17^ (GATK-3.5.0) and DV-GLx (DeepVariant v0.10.0, GLnexus version 1.2.6)-optimized pipeline^18,22,23^ were restricted to chromosome 20. Refinement of genotype likelihoods into hard genotype calls was performed with Beagle 4.1^27^ in approximate chunks of 1.4 Mbp (chunk size varied to keep the number of markers constant) with a 400 kbp overlap between each chunk. SHAPEIT-4.1.3 and Eagle-2.4.1 were then applied to the resulting hard genotypes in ~10 Mbp chunks with a 400 kbp overlap between each chunk. Chunks were ligated into whole chromosomes using bcftools^40^. Code for this analysis is included in our repository^41^ (Supplementary Table 7).

### GATK-3.5.0 reference panel creation

We applied GATK-3.5.0 best practices for joint calling, including recommended variant quality score recalibration (VQSR) thresholds. In addition to VQSR filtering, we removed singletons and variants where >10% of genotypes were missing. The resulting reference panel contained 36.1 million SNPs and 7.7 million indels across all autosomes. Based on the results in the previous section, we refined genotype likelihoods using Beagle 4.1 followed by phasing with SHAPEIT-4.1.3. See the script in Supplementary Table 7 for full details of the phasing pipeline.

### DV-GLx reference panel creation

The reference panel using DeepVariant-0.10.0 and GLnexus-1.2.6 was created independently of the GATK panel. We used results from a previous study on DV-GLx best practices^22^ to determine the optimal GLnexus merging parameters for ~15× coverage reads. After merging, we removed singletons and applied additional variant-level filters using (1) the Hardy-Weinberg equilibrium *p*-value (≥10^−20^), (2) the proportion of missing genotype calls in all samples (≤20%), and (3) the expected proportion of correct genotypes computed using Genotype Qualities (GQs) of all genotype calls (≥60%). Then, 43.7 million SNPs and 8.8 million indels were retained after filtering across the autosomes. Finally, we phased the variants with SHAPEIT-4.1.3 to generate the imputation reference panel. Notably, we did not perform genotype refinement with Beagle for DV-GLx, as it is computationally expensive and did not improve quality for DV-GLx calls. See Supplementary Methods for the specific commands used.

### WGS truth set used in imputation panel evaluations

We sought to create a fair truth set using high-coverage WGS data that could be imputed on the TOPMed imputation server. We extracted the 103 individuals who identified as AFAM from the GTEx V8 database^28^. For each individual, we took to the intersection of GATK-3.5 variants and DeepVariant-0.10.0 variants, then set genotypes where the variant callers disagreed or either caller had GQ < 20 to missing. Any resulting variants with >10% missing genotypes across the cohort of 103 samples were excluded. We only considered HG001/HG002/HG005 GIAB regions that were outside of segmental duplications. Variants were then lifted to hg19 using Picard to accommodate the older 1KGP/HRC/CAAPA panels. Only the set of successfully lifted variants were considered in the final evaluation (on both hg19 and GRCh38). Finally, to provide an objective estimate of AFAM allele frequency, we evaluated accuracy within frequency bins from gnomAD r3. This meant that the variant set was further limited to mutations present in gnomAD. The resulting truth set contained 21,642,652 SNPs and 1,939,396 indels.

### Microarray data used as truth in hap.py evaluations

We applied stringent filtering to create a high-quality truth set from 23andMe genotype microarray data for evaluating the performance of single-sample variant calling. It is noteworthy that these filters are much more stringent than what would typically be applied in a GWAS setting. In addition to typical filters on allele frequency and call rate, probes were aligned with BWA-MEM to ensure high specificity to the reference genome, and that the vendor-provided coordinates were consistent with alignment. We also excluded variants whose probes overlapped other common variants. This was due to the inability of probes to distinguish between certain alleles at multi-allelic sites and because a probe may fail to hybridize if it overlaps a flanking variant near the targeted variant.

The following filters were applied to a custom 23andMe genotype microarray (version 4):

- Located on autosome.
- ≥90% Call rate across entire research-consented database.
- ≥0.001% Minor allele frequency across the entire research-consented database.
- Minor allele count ≥ 1 within our cohort of 2,294 sequenced individuals.
- Probes aligned by BWA had Mapping Quality (MAPQ field) = 60, edit distance to reference (NM tag) = 0, and no clipping.
- BWA alignment agreed with vendor-provided coordinates.
- Entire 50-mer probe did not overlap any variant occurring in TOPMed with >1% MAF.
- Probes did not overlap one another on the chip.

The resulting truth set contained 387,493 SNPs and 73 indels. Genotypes from these variants were provided to hap.py as both a Variant Call Format (VCF) file (for non-reference genotypes) and a confident region Browser-Extensible Data (BED) file (derived from both homozygous reference and non-reference genotypes) for evaluation of single-sample variant calling.

### Reporting summary

Further information on research design is available in the Nature Research Reporting Summary linked to this article.

## Supplementary information

Supplementary Information

Description of Additional Supplementary Files

Supplementary Data 1

Reporting Summary

## Data Availability

The imputation panel and associated sequencing data described here are available on dbGaP (phs001798.v2.p2) for Human Genetic Variation Research. Raw data underlying all main text figures, except Fig. 1a, are available in Supplementary Data 1. Other individual-level data from 23andMe participants used in the analyses are not publicly available due to participant confidentiality and in accordance with the IRB-approved protocol under which the study was conducted. Aggregate-level data will be made available on reasonable request to dataset-request@23andme.com.

## Code availability

Analysis scripts are available at: 10.5281/zenodo.5527247^41^.
