# Supplementary material for: A population-specific reference panel for improved genotype imputation in African Americans
Source: Commun Biol. 2021 Nov 5;4:1269. doi: 10.1038/s42003-021-02777-9 (PMC8571350; doi:10.1038/s42003-021-02777-9)
Supplement: Supplementary file 1 — Supplementary Information [file 42003_2021_2777_MOESM1_ESM.pdf]

Supplementary Information - A population-specific reference panel for improved genotype imputation in African Americans

Jared O'Connell[1,*], Taedong Yun[2,*], Meghan Moreno[1], Helen Li[2], Nadia Litterman[1], Alexey Kolesnikov[2], Elizabeth Noblin[1], Pi-Chuan Chang[2], Anjali Shastri[1], Elizabeth H. Dorfman[2], Suyash Shringarpure[1], 23andMe Research Team[‡], Adam Auton[1,^], Andrew Carroll[2,†,^], Cory Y. McLean[2,†,^]

[1]23andMe Inc., Sunnyvale, CA, USA.
[2]Google, Health, Cambridge, MA, USA and Palo Alto, CA, USA.
* These authors contributed equally.
† These authors jointly supervised this work.
^ To whom correspondence should be addressed: aauton@23andme.com (A.A.), awcarroll@google.com (A.C.), cym@google.com (C.Y.M.).
‡ A full list of the 23andMe Research Team members appears at the end of the main text.

Supplementary Methods 2

Supplementary Figures 4

Supplementary Tables 10

Supplementary References 18

Supplementary Methods

DeepVariant-GLnexus reference panel creation

We applied DeepVariant-0.10.0 in its default settings to generate single-sample AFAM gVCF files and merged them with GLnexus using parameters optimized for ~15X coverage in a previous study[1].

For autosomes, the following Bash script describes our complete pipeline for generating a reference panel from AFAM cohort variant calls by DeepVariant-GLnexus. It requires bcftools[2] (version 1.10.2-105-g7cd83b7 or later), SHAPEIT[3] (version 4.1.3), tabix[4], and the custom "`expected-correct`" bcftools plugin available from the accompany code repository[1].

```
# For chromosome 1 as an example - the same commands for other chromosomes.
CHROM="chr1"

# The genetic map file can be downloaded from SHAPEIT4 GitHub.
GENETIC_MAP="${CHROM}.b38.gmap.gz"

# Inputs
COHORT_VCF="cohort-${CHROM}.vcf.gz"
REF_GENOME="GRCh38_reference_genome.fa"

# Intermediate outputs
FILTERED_VCF="cohort-filtered-${CHROM}.vcf.gz"
PHASED_VCF="cohort-phased-${CHROM}.vcf.gz"
PHASED_ANNOTATED_VCF="cohort-phased-annotated-${CHROM}.vcf.gz"
ANNOTATION_FILE="annotation-${CHROM}.tab.gz"
ANNOTATION_HEADER_FILE="annotation-header.txt"

# Final output VCF (the reference panel)
REFERENCE_PANEL_VCF="reference-panel-${CHROM}.vcf.gz"

# Filtering and normalizing before phasing
bcftools view ${COHORT_VCF} -Ou -f ".,PASS" | \
  bcftools norm -Ou -m -any | \
  bcftools view -Ou -c 2 | \
  bcftools norm -d none -f ${REF_GENOME} -Oz -o ${FILTERED_VCF}
bcftools index -t ${FILTERED_VCF}

# Save annotations in a separate file before phasing,
# because SHAPEIT4 will remove all annotations.
bcftools +fill-tags ${FILTERED_VCF} -Ou -- -t F_MISSING | \
  bcftools +expected-correct -Ou | \
  bcftools query -f '%CHROM\t%POS\t%REF\t%ALT\t%F_MISSING\t%EXPECTED_CORRECT\n' | \
    bgzip -c > ${ANNOTATION_FILE}
tabix -s1 -b2 -e2 ${ANNOTATION_FILE}

# Phasing
shapeit4 \
```

[1]https://github.com/23andme-jaredo/african-american-sequencing-paper/blob/main/bcftools-plugins/expected-correct.c

```
  --input ${FILTERED_VCF} \
  --output ${PHASED_VCF} \
  --map ${GENETIC_MAP} \
  --region ${CHROM} \
  --thread $(nproc) \
  --pbwt-depth 8 \
  --pbwt-mdr 0.01 \
  --mcmc-iterations "10b,1p,1b,1p,1b,1p,1b,1p,10m" \
  --sequencing
bcftools index -t ${PHASED_VCF}

bcftools +fill-tags ${PHASED_VCF} -Oz -o ${PHASED_ANNOTATED_VCF}
bcftools index -t ${PHASED_ANNOTATED_VCF}

# Create annotation header
echo '##INFO=<ID=ORIG_F_MISSING,Number=1,Type=Float,Description="Fraction of
missing genotypes before phasing">' > ${ANNOTATION_HEADER_FILE}
echo '##INFO=<ID=EXPECTED_CORRECT,Number=1,Type=Float,Description="Expected
proportion of correct genotypes before phasing">' >> ${ANNOTATION_HEADER_FILE}

# Add annotation from the saved annotation file,
# and filter variants using the annotation.
bcftools annotate ${PHASED_ANNOTATED_VCF} -Ou \
  -a ${ANNOTATION_FILE} \
  -h ${ANNOTATION_HEADER} \
  -c CHROM,POS,REF,ALT,ORIG_F_MISSING,EXPECTED_CORRECT | \
  bcftools view -Oz -o ${REFERENCE_PANEL_VCF} \
    -i "HWE >= 1e-20 && ORIG_F_MISSING <= 0.2 && EXPECTED_CORRECT >= 0.6"

bcftools index -t ${REFERENCE_PANEL_VCF}
```

For chromosome X, we found that SHAPEIT4 fails during the HMM computation step when applied to the whole chromosome X cohort. We worked around this issue by sharding the VCF into 8 shards with 400Kbp overlaps (`"chunker.py -w 20000000 -b 200000"`)[2], phasing each shard with SHAPEIT4, and then ligating the phased shards while matching the phase in overlapping regions (`"bcftools concat -lc"`)[3]. Then we followed the same annotation and filtering steps as above, except that we omitted the `"HWE >= 1e-20"` filter for chromosome X.

[2] https://github.com/23andme-jaredo/african-american-sequencing-paper/blob/main/src/python/chunker.py
[3] http://samtools.github.io/bcftools/bcftools.html#concat

Supplementary Figures

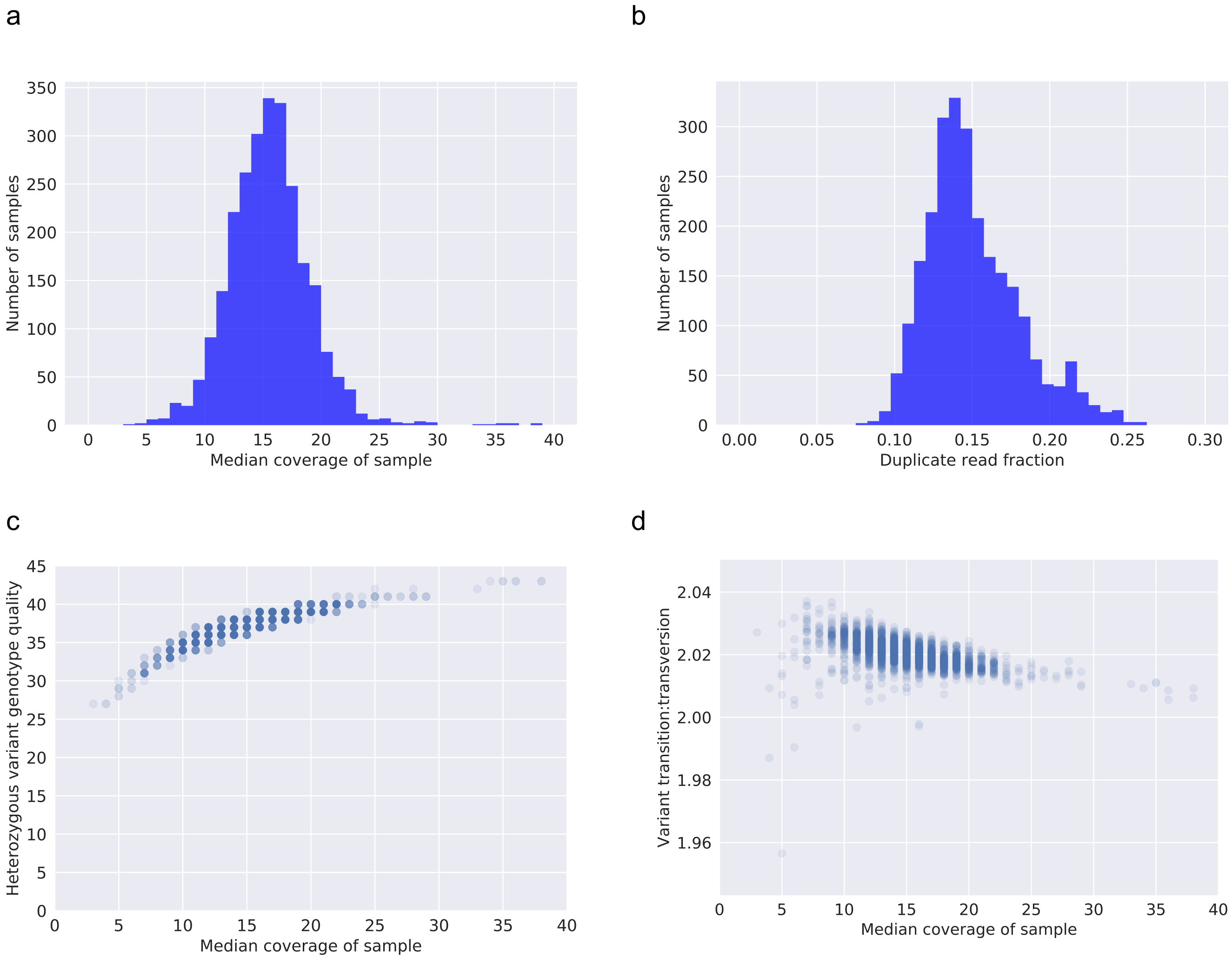

Supplementary Figure 1. Sample quality metrics. a) Histogram of the median coverage for samples in the cohort, determined as uniquely mapped coverage of genome bases. **b)** Histogram of the fraction of reads marked as duplicates by Picard MarkDuplicates for samples in the cohort. **c)** The median genotype quality of heterozygous variant calls as reported by DeepVariant (representing its confidence in the call) as a function of the coverage of the sample. **d)** The transition:transversion ratio of calls in a sample as a function of coverage.

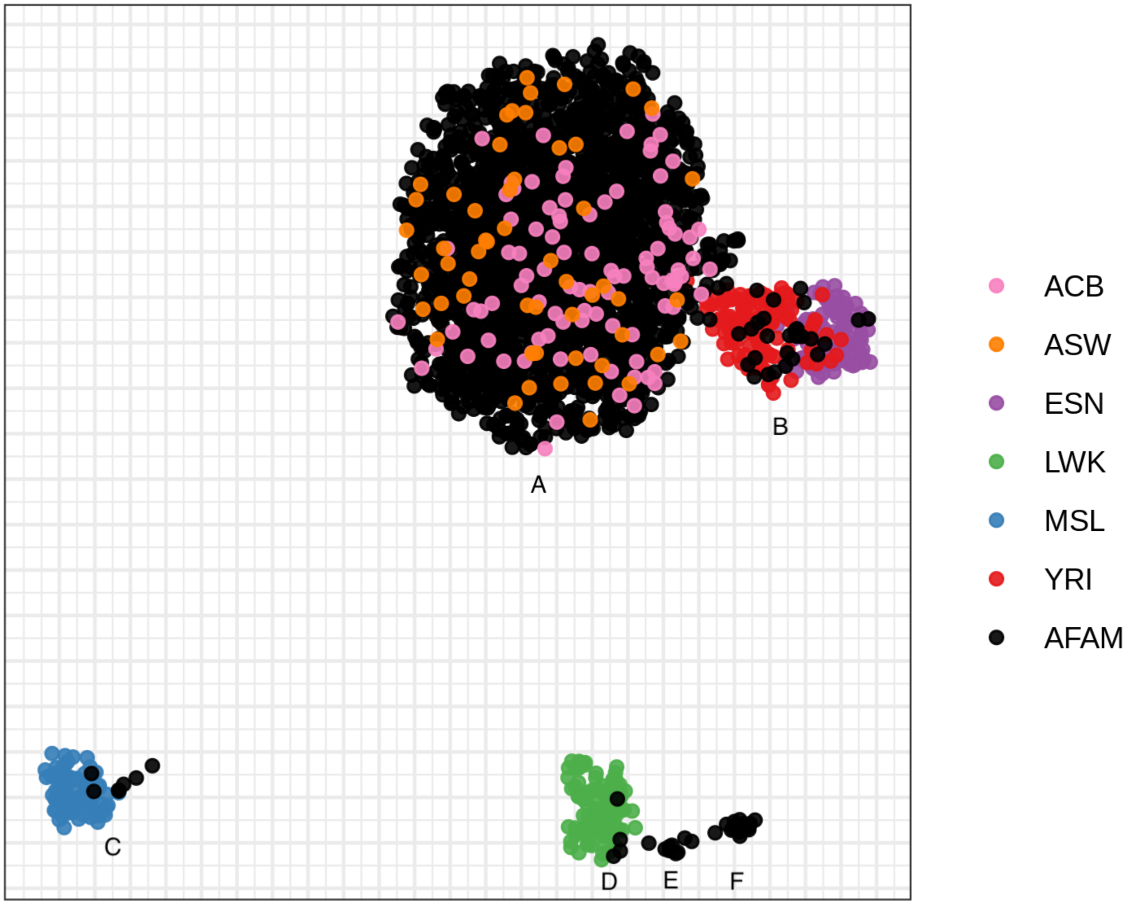

Supplementary Figure 2: UMAP analysis of AFAM samples and relevant 1KGP sub-populations. We performed UMAP on AFAM samples and six relevant 1KGP populations using the first 15 principal components. The vast majority of AFAM samples clusters with the ASW (African Ancestry SW) and ACB (African Caribbean) groups from 1KGP. With small numbers clustering with YRI/ESN (Yoruba in Ibadan, Nigeria/Esan in Nigeria), MSL (Mende in Sierra Leone) and LWK (Luhya in Webuye, Kenya). We manually curated six clusters labelled A through F, and we report the estimated ancestry proportions from 23andMe's classifier in **Supplementary Table 4**.

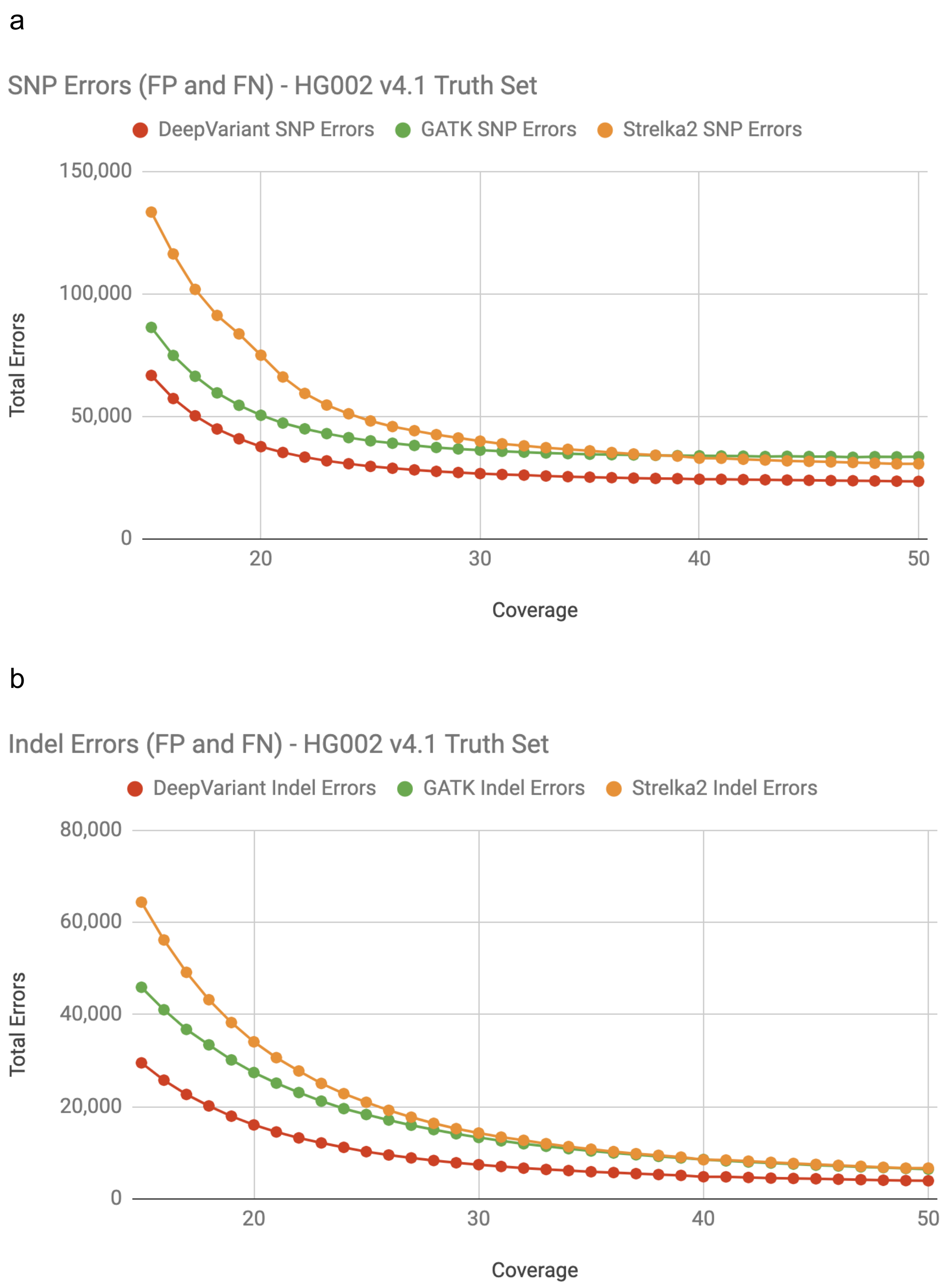

Supplementary Figure 3: Coverage analysis of SNP and indel accuracy on germline variant calling pipelines. a) Total SNP errors (false positives plus false negatives) in HG002 in the Genome in a Bottle v4.1 truth regions as a function of sequence coverage for DeepVariant 0.10.0, GATK 4.1.0.0, and Strelka 2.9.10. Lower is better. **b**) Same as in **a)**, but for indels.

a

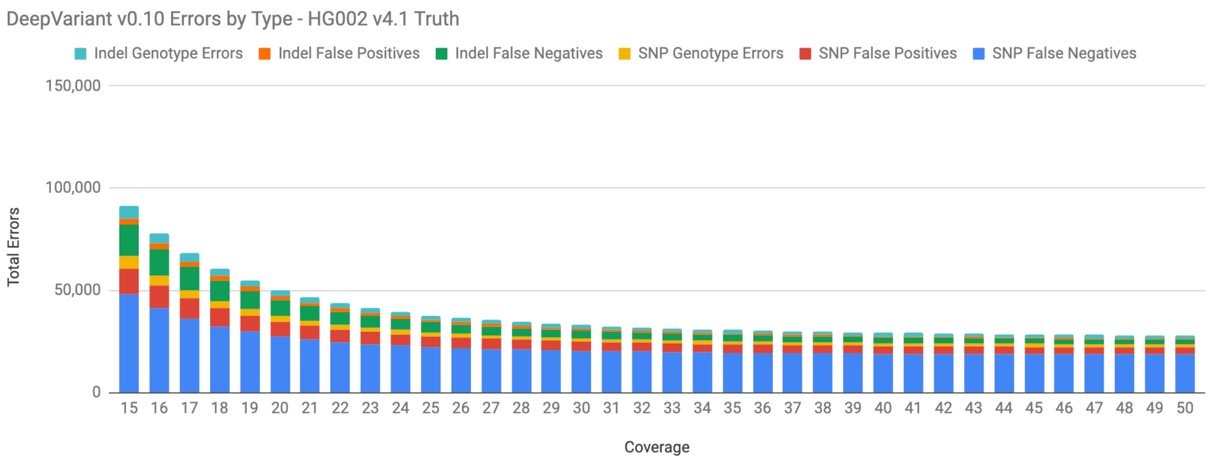

b

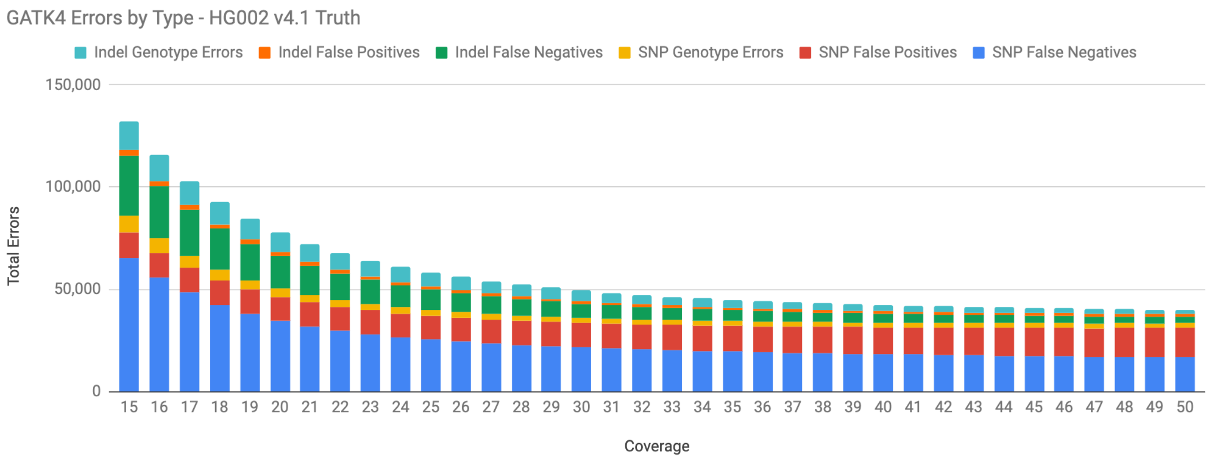

Supplementary Figure 4: Analysis of variant calling errors by type for DeepVariant and GATK4 at different sequence coverages. a) Errors in HG002 in the Genome in a Bottle v4.1 truth regions, annotated by error type (false positives, false negatives, or genotype errors (miscalling a heterozygous event as homozygous or vice-versa), separately for SNPs and indels) as a function of sequence coverage for DeepVariant 0.10.0. **b**) Same as in **a)**, but for GATK 4.1.0.0.

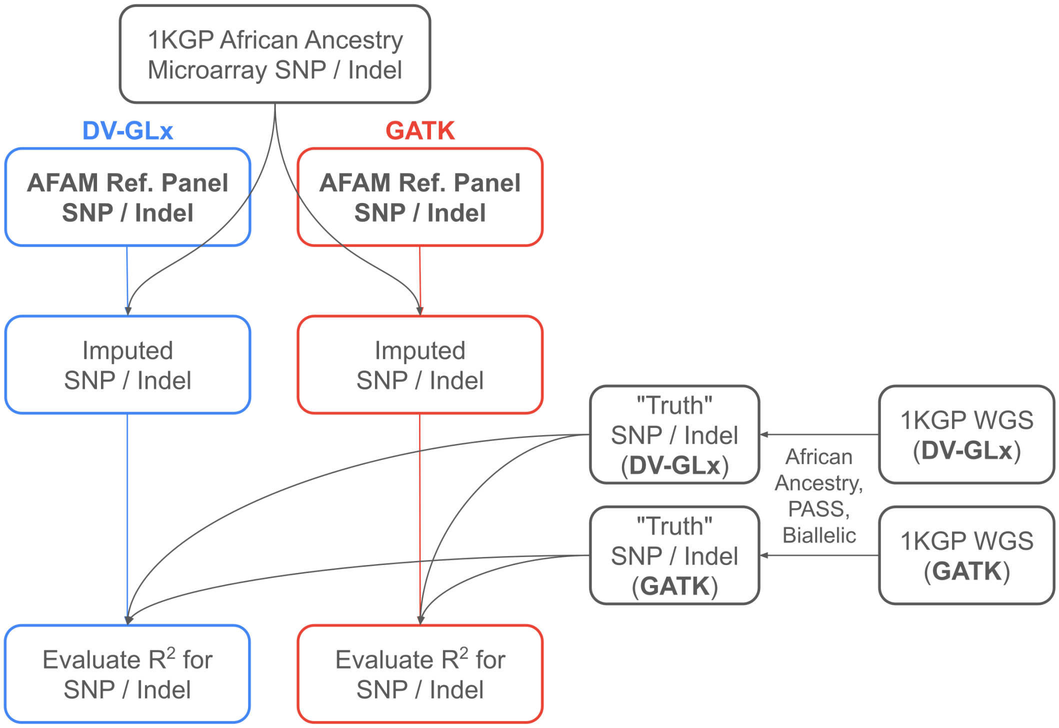

Supplementary Figure 5: Imputation panel evaluation workflow. For evaluating R^2, the imputed genotypes were binned by alternate allele frequency computed in the 1KGP WGS cohort (the same one used for generating the "truth" variants) with all 2,504 samples included. See also **Figure 3**. Ref: reference, 1KGP: the 1000 Genomes Project, DV-GLx: DeepVariant-GLnexus, WGS: whole-genome sequencing.

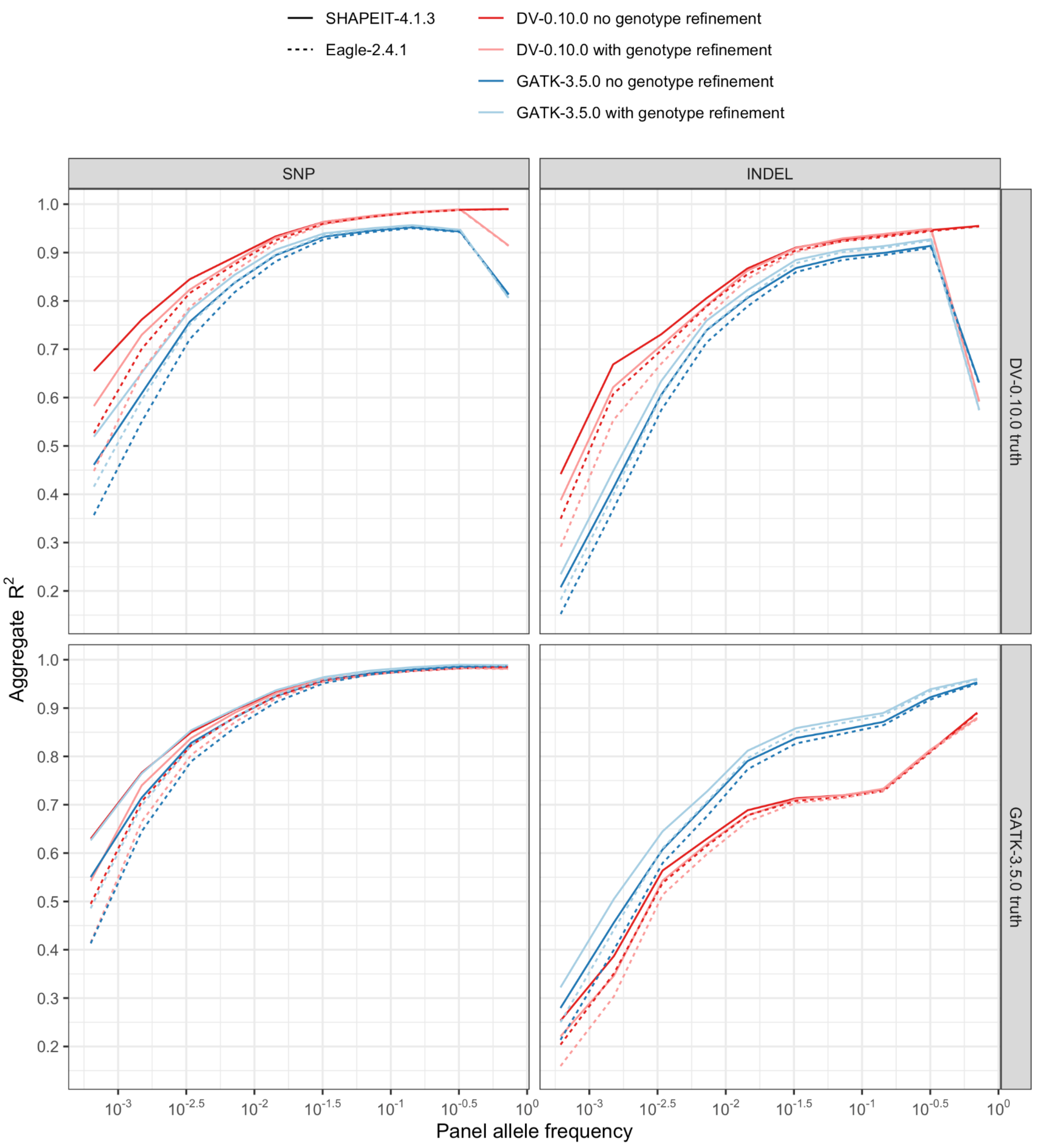

Supplementary Figure 6: Imputation panel performance as a function of variant calling pipeline, inclusion of genotype refinement, and phasing algorithm. We evaluated performance on chr20 using two truth sets created with different variant callers on the same set of 240 African samples from 1KGP. If a variant in the truth set was missing in an imputation panel it was treated as if being imputed to homozygous reference in the truth set, hence penalizing panels that are missing variants.

Supplementary Tables

Supplementary Table 1: Sample size and variant counts for six popular imputation panels. Indels denote any non-SNP variant in these counts.

Name	Sample count	SNP count	Indel count	Download available
1000 Genomes phase 3 (1KGP) *	2,504	45,568,747	3,438,564	Yes
Haplotype Reference Consortium (HRC) **	27,166	40,405,505	0	Yes
AFAM	2,269	45,802,366	9,160,064	Yes
Consortium on Asthma among African-ancestry Populations in the Americas (CAAPA) ***	883	29,850,179	0	Yes
African Genome Resources (AGR)	4,956	93,421,145	0	No
NHLBI Trans-Omics for Precision Medicine (TOPMed)	97,256	286,039,888	22,032,924	No

* Singletons and duplicated variants were filtered from 1000 Genomes in these counts.
** This is the downloadable version available on ENA which is slightly smaller than the server version.
*** chrX is not available for this panel.

Supplementary Table 2: Reported country of birth for all sequenced participants. Participants reported residing in the USA when re-contacted. Counts fewer than six are masked for anonymity reasons.

Region	Count
North America - US	1,689
North America - Caribbean	74
Africa	37
Europe	31
Asia	<6
South America	<6
North America - CA	<6
Oceania	<6
not reported	451
Total	2,294

Supplementary Table 3: Counts of African populations used in 23andMe's ancestry classifier. "23andMe" refers to 23andMe customers used in the classifier and "Public" refers to individuals from publicly available datasets.

Population	23andMe	Public	Total
Biaka, Mbuti & San	0	41	41
Congo	597	0	597
Ethiopia & Eritrea	171	0	171
Nigeria	54	226	280
Senegal, The Gambia & Guinea	23	135	158
Sierra Leone, Liberia, Ivory Coast & Ghana	196	85	281
Somalia	150	0	150
Southern East Africa	15	109	124
Sudan	189	0	189
**Total**	1,395	596	1,991

Supplementary Table 4: Estimated ancestry proportions for the six manually curated clusters from **Supplementary Figure 2**. The ancestry proportions for each cluster of our cohort broadly agree with the 1KGP individuals with whom they group. Individuals in cluster **A** are of admixed Atlantic African, European and American descent and group with ASW/ACB. Individuals in cluster **B** are largely of (modern day) Nigerian descent and group with YRI/ESL. Individuals in cluster **C** are of Sierra Leone, Liberia, Ivory Coast & Ghana descent and group with MSL. Finally, individuals in clusters **D**, **E**, and **F** are of South and North Eastern African descent. **D** groups tightly with LWK while **E** and **F** do not appear to cluster closely with any 1KGP ethnicities, likely due to their ancestries not being well represented in the 1KGP cohort.

Region	Sub Region	A	B	C	D	E	F
Atlantic Africa	Senegal, The Gambia & Guinea	6.4	0.5	7.9	0.1	0.1	0.0
Atlantic Africa	Broadly Atlantic Africa	9.0	3.0	3.6	1.4	1.0	0.1
Atlantic Africa	Sierra Leone, Liberia, Ivory Coast & Ghana	20.8	7.4	81.0	0.3	0.1	0.0
Atlantic Africa	Nigeria	31.1	85.0	5.1	1.4	0.6	0.0
European	European	15.8	0.5	0.4	0.2	0.1	0.1
Congo & Southern East Africa	Broadly Congo & Southern East Africa	2.1	0.4	0.2	5.5	1.9	0.0
Congo & Southern East Africa	Congo	7.9	2.1	0.6	25.6	1.6	0.0
Congo & Southern East Africa	Southern East Africa	0.9	0.1	0.1	56.0	46.4	0.0
Broadly African	Broadly African	3.2	0.7	0.6	3.4	2.4	0.2
Native American/East Asian	Native American/East Asian	1.2	0.0	0.1	0.1	0.1	0.0
Northern East Africa	Broadly Northern East Africa	0.0	0.0	0.0	0.5	1.0	0.4
Northern East Africa	Somalia	0.0	0.0	0.0	0.0	0.0	32.8
Northern East Africa	Sudan	0.1	0.0	0.0	3.5	32.4	0.9
Northern East Africa	Ethiopia & Eritrea	0.0	0.0	0.0	0.6	11.5	64.8
Biaka, Mbuti & San	Biaka, Mbuti & San	0.3	0.1	0.0	0.5	0.1	0.0
Other/Uncertain	Other/Uncertain	1.1	0.1	0.2	0.9	0.7	0.7

Supplementary Table 5: Total discordance and non-reference discordance for imputed genotypes for each panel/method combination. Truth data were 103 individuals from GTEx[5] that self-identified as African American.

Method	Panel	Variant	Non Reference Discordance (%)	Discordance (%)
Beagle-5.1	AFAM-DV-GLx	SNP	11.56	2.22
Minimac-4	AFAM-DV-GLx	SNP	13.13	2.51
Minimac-4	TOPMed	SNP	8.34	1.5
Beagle-5.1	1KGP	SNP	13.13	2.69
Minimac-4	1KGP	SNP	14.3	2.92
Beagle-5.1	HRC	SNP	14.9	3.22
Minimac-4	HRC	SNP	16.05	3.47
Minimac-4	CAAPA	SNP	21.25	4.35
Beagle-5.1	AFAM-DV-GLx	INDEL	12.57	2.35
Minimac-4	AFAM-DV-GLx	INDEL	14.25	2.64
Minimac-4	TOPMed	INDEL	9.27	1.45
Beagle-5.1	1KGP	INDEL	14.34	3.09
Minimac-4	1KGP	INDEL	15.53	3.34

Supplementary Table 6: Confusion matrices for every combination of imputation panel and variant caller, stratified by variant type. These values were used to calculate the discordance metrics in Supplementary Table 5.

				Estimated genotype			
**Method**	**Panel**	**Variant**	**Truth Genotype**	**0/0**	**0/1**	**1/1**	**./.**
Beagle-5.1	AFAM-DV-GLx	SNP	0/0	1,622,244,368	12,062,248	244,653	200,238,827
Beagle-5.1	AFAM-DV-GLx	SNP	0/1	22,720,892	227,221,509	4,777,161	2,265,957
Beagle-5.1	AFAM-DV-GLx	SNP	1/1	407,269	4,464,267	114,742,073	179,484
Beagle-5.1	AFAM-DV-GLx	INDEL	0/0	144,414,073	1,143,507	23,410	16,815,651
Beagle-5.1	AFAM-DV-GLx	INDEL	0/1	2,154,073	19,730,331	424,807	198,008
Beagle-5.1	AFAM-DV-GLx	INDEL	1/1	34,514	391,926	9,283,025	20,603
Minimac-4	AFAM-DV-GLx	SNP	0/0	1,624,488,423	9,855,790	207,056	200,238,827
Minimac-4	AFAM-DV-GLx	SNP	0/1	29,600,928	220,255,304	4,863,330	2,265,957
Minimac-4	AFAM-DV-GLx	SNP	1/1	634,628	5,320,084	113,658,897	179,484
Minimac-4	AFAM-DV-GLx	INDEL	0/0	144,628,277	933,364	19,349	16,815,651
Minimac-4	AFAM-DV-GLx	INDEL	0/1	2,797,516	19,084,045	427,650	198,008
Minimac-4	AFAM-DV-GLx	INDEL	1/1	51,254	468,187	9,190,024	20,603
Minimac-4	TOPMed	SNP	0/0	1,738,274,547	8,251,368	129,649	88,134,532
Minimac-4	TOPMed	SNP	0/1	16,260,399	233,734,430	3,388,869	3,601,821
Minimac-4	TOPMed	SNP	1/1	279,444	3,394,448	114,885,973	1,233,228
Minimac-4	TOPMed	INDEL	0/0	118,493,786	533,524	7,281	43,362,050
Minimac-4	TOPMed	INDEL	0/1	1,092,274	14,215,990	189,617	7,009,338
Minimac-4	TOPMed	INDEL	1/1	18,176	193,431	5,705,231	3,813,230
Beagle-5.1	1KGP	SNP	0/0	1,499,396,657	14,268,132	316,900	320,853,342
Beagle-5.1	1KGP	SNP	0/1	24,913,432	221,885,585	5,382,468	4,804,034
Beagle-5.1	1KGP	SNP	1/1	501,144	5,236,407	112,971,618	1,038,989
Beagle-5.1	1KGP	INDEL	0/0	107,584,081	1,195,105	48,023	53,569,529
Beagle-5.1	1KGP	INDEL	0/1	2,043,614	17,045,054	446,852	2,971,745
Beagle-5.1	1KGP	INDEL	1/1	54,803	440,280	8,205,667	1,029,442
Minimac-4	1KGP	SNP	0/0	1,499,878,812	13,804,927	297,950	320,853,342
Minimac-4	1KGP	SNP	0/1	28,538,432	218,033,631	5,609,422	4,804,034
Minimac-4	1KGP	SNP	1/1	614,310	6,178,748	111,916,111	1,038,989
Minimac-4	1KGP	INDEL	0/0	107,625,368	1,155,737	46,104	53,569,529
Minimac-4	1KGP	INDEL	0/1	2,330,621	16,743,673	461,226	2,971,745
Minimac-4	1KGP	INDEL	1/1	63,197	516,197	8,121,356	1,029,442
Beagle-5.1	HRC	SNP	0/0	1,331,046,240	17,158,029	397,148	486,233,614
Beagle-5.1	HRC	SNP	0/1	25,060,018	209,059,345	5,745,592	17,120,564

Beagle-5.1	HRC	SNP	1/1	556,816	5,785,628	103,442,500	9,963,214
Beagle-5.1	HRC	INDEL	0/0	0	0	0	162,396,738
Beagle-5.1	HRC	INDEL	0/1	0	0	0	22,507,265
Beagle-5.1	HRC	INDEL	1/1	0	0	0	9,730,192
Minimac-4	HRC	SNP	0/0	1,331,334,704	16,899,864	366,849	486,233,614
Minimac-4	HRC	SNP	0/1	28,075,583	205,616,236	6,173,136	17,120,564
Minimac-4	HRC	SNP	1/1	637,076	6,751,943	102,395,925	9,963,214
Minimac-4	HRC	INDEL	0/0	0	0	0	162,396,738
Minimac-4	HRC	INDEL	0/1	0	0	0	22,507,265
Minimac-4	HRC	INDEL	1/1	0	0	0	9,730,192
Minimac-4	CAAPA	SNP	0/0	1,463,040,164	12,776,101	502,452	358,516,314
Minimac-4	CAAPA	SNP	0/1	49,115,378	189,973,466	7,665,637	10,231,038
Minimac-4	CAAPA	SNP	1/1	2,211,769	7,704,228	106,374,938	3,457,223
Minimac-4	CAAPA	INDEL	0/0	0	0	0	162,396,738
Minimac-4	CAAPA	INDEL	0/1	0	0	0	22,507,265
Minimac-4	CAAPA	INDEL	1/1	0	0	0	9,730,192

Supplementary Table 7: Scripts used for the various pipelines we applied to create the imputation panels described in this paper. Nextflow[6] script locations are relative to the public GitHub repository https://github.com/23andme-jaredo/african-american-sequencing-paper.

Description	Script
Produces four panels: with Beagle genotype refinement/no refinement and Eagle2/SHAPEIT4 phasing.	`src/nf/phasing-and-refinement-experiments.nf`
Beagle genotype refinement followed by SHAPEIT4 phasing used for GATK panel.	`src/nf/beagle-shapeit.nf`

Supplementary References

1. Yun, T. *et al.* Accurate, scalable cohort variant calls using DeepVariant and GLnexus. *Bioinformatics* **36**, 5582–5589 (2020).
2. Danecek, P. *et al.* Twelve years of SAMtools and BCFtools. *GigaScience* **10**, giab008 (2021).
3. Delaneau, O., Zagury, J.-F., Robinson, M. R., Marchini, J. L. & Dermitzakis, E. T. Accurate, scalable and integrative haplotype estimation. *Nat. Commun.* **10**, 5436 (2019).
4. Li, H. Tabix: fast retrieval of sequence features from generic TAB-delimited files. *Bioinformatics* **27**, 718–719 (2011).
5. The GTEx Consortium. The GTEx Consortium atlas of genetic regulatory effects across human tissues. *Science* **369**, 1318–1330 (2020).
6. Di Tommaso, P. *et al.* Nextflow enables reproducible computational workflows. *Nat. Biotechnol.* **35**, 316–319 (2017).
