# Supplementary material for: A population-specific reference panel for improved genotype imputation in African Americans
Source: Commun Biol. 2021 Nov 5;4:1269. doi: 10.1038/s42003-021-02777-9 (PMC8571350; doi:10.1038/s42003-021-02777-9)
Supplement: Supplementary file 2 — Description of Additional Supplementary Files [file 42003_2021_2777_MOESM2_ESM.pdf]

Description of Additional Supplementary Files

File name: Supplementary Data 1

Description: Source data underlying all main text figure panels except Figure 1a. Note: Figure 1a data is omitted owing to consent and privacy issues documented more fully in the Data Availability Statement below.
