# Supplementary material for: A population-specific reference panel for improved genotype imputation in African Americans
Source: Commun Biol. 2021 Nov 5;4:1269. doi: 10.1038/s42003-021-02777-9 (PMC8571350; doi:10.1038/s42003-021-02777-9)
Supplement: Supplementary file 4 — Reporting Summary [file 42003_2021_2777_MOESM4_ESM.pdf]

nature portfolio

Corresponding author(s): Cory McLean

Last updated by author(s): Sep 25, 2021

# Reporting Summary

Nature Portfolio wishes to improve the reproducibility of the work that we publish. This form provides structure for consistency and transparency in reporting. For further information on Nature Portfolio policies, see our Editorial Policies and the Editorial Policy Checklist.

## Statistics

For all statistical analyses, confirm that the following items are present in the figure legend, table legend, main text, or Methods section.

| n/a | Confirmed | |
|---|---|---|
| ☐ | ☒ | The exact sample size (*n*) for each experimental group/condition, given as a discrete number and unit of measurement |
| ☐ | ☒ | A statement on whether measurements were taken from distinct samples or whether the same sample was measured repeatedly |
| ☐ | ☒ | The statistical test(s) used AND whether they are one- or two-sided<br>*Only common tests should be described solely by name; describe more complex techniques in the Methods section.* |
| ☒ | ☐ | A description of all covariates tested |
| ☒ | ☐ | A description of any assumptions or corrections, such as tests of normality and adjustment for multiple comparisons |
| ☒ | ☐ | A full description of the statistical parameters including central tendency (e.g. means) or other basic estimates (e.g. regression coefficient) AND variation (e.g. standard deviation) or associated estimates of uncertainty (e.g. confidence intervals) |
| ☒ | ☐ | For null hypothesis testing, the test statistic (e.g. *F*, *t*, *r*) with confidence intervals, effect sizes, degrees of freedom and *P* value noted<br>*Give P values as exact values whenever suitable.* |
| ☒ | ☐ | For Bayesian analysis, information on the choice of priors and Markov chain Monte Carlo settings |
| ☒ | ☐ | For hierarchical and complex designs, identification of the appropriate level for tests and full reporting of outcomes |
| ☐ | ☒ | Estimates of effect sizes (e.g. Cohen's *d*, Pearson's *r*), indicating how they were calculated |

*Our web collection on statistics for biologists contains articles on many of the points above.*

## Software and code

Policy information about availability of computer code

| | |
|---|---|
| Data collection | No software was used for data collection. |
| Data analysis | Custom scripts: https://github.com/23andme-jaredo/african-american-sequencing-paper<br>Read alignment: BWA-MEM 0.7.16a-r1181<br>Duplicate read marking: Picard 2.1.0<br>Variant calling: GATK 3.5.0, DeepVariant 0.10.0, GLnexus 1.2.6, GATK 4.1.0.0, Strelka 2.9.10<br>IBD and kinship analysis: AKT 0.3.2<br>Genotype refinement: Beagle 4.1<br>Phasing: SHAPEIT 4.1.3, bcftools 1.10.2-105-g7cd83b7<br>Imputation: Minimac 4, Beagle 5.1<br>Variant evaluation: hap.py 0.3.12 |

For manuscripts utilizing custom algorithms or software that are central to the research but not yet described in published literature, software must be made available to editors and reviewers. We strongly encourage code deposition in a community repository (e.g. GitHub). See the Nature Portfolio guidelines for submitting code & software for further information.

# Data

Policy information about availability of data

All manuscripts must include a data availability statement. This statement should provide the following information, where applicable:

- Accession codes, unique identifiers, or web links for publicly available datasets
- A description of any restrictions on data availability
- For clinical datasets or third party data, please ensure that the statement adheres to our policy

The imputation panel and associated sequencing data described here are available on dbGaP (phs001798.v2.p2) for Human Genetic Variation Research. Raw data underlying all main text figures except Figure 1a are available in Supplementary Data 1. Other individual-level data from 23andMe participants used in the analyses are not publicly available due to participant confidentiality, and in accordance with the IRB-approved protocol under which the study was conducted. Aggregate-level data will be made available on reasonable request to dataset-request@23andme.com.

# Field-specific reporting

Please select the one below that is the best fit for your research. If you are not sure, read the appropriate sections before making your selection.

☒ Life sciences ☐ Behavioural & social sciences ☐ Ecological, evolutionary & environmental sciences

For a reference copy of the document with all sections, see nature.com/documents/nr-reporting-summary-flat.pdf

# Life sciences study design

All studies must disclose on these points even when the disclosure is negative.

| | |
|---|---|
| Sample size | 2,269 samples of African American genetic ancestry were compiled into a final imputation reference panel. This was an attempt to maximize sample size at a fixed project budget. |
| Data exclusions | We re-contacted 71,455 23andMe customers who met the following criteria: had consented to participate in 23andMe research, identified as having African ancestry, were over 18 years of age, joined 23andMe after 2010, had answered >100 survey questions and who were residing in the USA. From this pool of re-contacted candidates, 5,404 individuals further consented to have their individual-level sequencing data made available via dbGaP. We then sequenced the 2,294 individuals with the highest amount of estimated Sub-Saharan African ancestry to produce the final cohort. Finally we uploaded their sequence data to dbGaP (after removing QC failures). The final reference panel also removed close relatives, leaving 2,269 unrelated samples in the panel. |
| Replication | Performance of the panel for imputation of samples with African ancestry was performed in both 1000 Genomes samples of African ancestry and GTEx samples of self-reported African-American ancestry. |
| Randomization | Not relevant -- this paper is about generating a single imputation reference panel for improving imputation of African-American individuals. |
| Blinding | Not relevant -- this paper is about generating a single imputation reference panel for improving imputation of African-American individuals. |

# Reporting for specific materials, systems and methods

We require information from authors about some types of materials, experimental systems and methods used in many studies. Here, indicate whether each material, system or method listed is relevant to your study. If you are not sure if a list item applies to your research, read the appropriate section before selecting a response.

## Materials & experimental systems

| n/a | Involved in the study | |
|---|---|---|
| ☒ | ☐ | Antibodies |
| ☒ | ☐ | Eukaryotic cell lines |
| ☒ | ☐ | Palaeontology and archaeology |
| ☒ | ☐ | Animals and other organisms |
| ☐ | ☒ | Human research participants |
| ☒ | ☐ | Clinical data |
| ☒ | ☐ | Dual use research of concern |

## Methods

| n/a | Involved in the study | |
|---|---|---|
| ☒ | ☐ | ChIP-seq |
| ☒ | ☐ | Flow cytometry |
| ☒ | ☐ | MRI-based neuroimaging |

## Human research participants

Policy information about studies involving human research participants

| | |
|---|---|
| Population characteristics | The relevant characteristics are that all samples have high African ancestry content in their genome, were over 18 years of age, and reside in the USA. |
| Recruitment | We re-contacted 71,455 23andMe customers who met the following criteria: had consented to participate in 23andMe research, identified as having African ancestry, were over 18 years of age, joined 23andMe after 2010, had answered >100 survey questions and who were residing in the USA. From this pool of re-contacted candidates, 5,404 individuals further consented to have their individual-level sequencing data made available via dbGaP. We then sequenced the 2,294 individuals with the highest amount of estimated Sub-Saharan African ancestry to produce the final cohort. |
| Ethics oversight | Ethical & Independent (E&I) Review Services IRB. |

Note that full information on the approval of the study protocol must also be provided in the manuscript.
